# Theoretical, thermodynamic and electrochemical analysis of biotin drug as an impending corrosion inhibitor for mild steel in 15% hydrochloric acid

**DOI:** 10.1098/rsos.170933

**Published:** 2017-12-06

**Authors:** Xihua Xu, Ambrish Singh, Zhipeng Sun, K. R. Ansari, Yuanhua Lin

**Affiliations:** 1 School of Materials Science and Engineering, Southwest Petroleum University, Chengdu, Sichuan 610500, China; 2 State Key Laboratory of Oil and Gas Reservoir Geology and Exploitation, Southwest Petroleum University, Chengdu, Sichuan 610500, China; 3 Department of Applied Chemistry, Indian Institute of Technology (Banaras Hindu University), Varanasi 221005, India

**Keywords:** biotin, SECM, AFM, electrochemical, corrosion inhibitor, quantum calculations

## Abstract

The corrosion mitigation efficiency of biotin drug for mild steel in 15% hydrochloric acid was thoroughly investigated by weight loss and electrochemical methods. The surface morphology was studied by the contact angle, scanning electrochemical microscopy, atomic force microscopy and scanning electron microscopy methods. Quantum chemical calculation and Fukui analysis were done to correlate the experimental and theoretical data. The influence of the concentration of inhibitor, immersion time, temperature, activation energy, enthalpy and entropy has been reported. The mitigation efficiency of biotin obtained by all methods was in good correlation with each other. Polarization studies revealed that biotin acted as a mixed inhibitor. The adsorption of biotin was found to obey the Langmuir adsorption isotherm. Surface studies showed the hydrophobic nature of the steel with inhibitor and vindicated the formation of a film on the metal surface that reduced the corrosion rate.

## 1. Introduction

Mild steel is used in acidization and pickling industries owing to its cost-effectiveness. During the acidization and pickling processes the steel is prone to corrosion owing to the presence of acids. Use of inhibitors is a common method to mitigate corrosion owing to its ease of availability, effectiveness and cheapness. A good inhibitor should follow the regulations of environment safety and be eco-friendly. A number of compounds have been reported as corrosion inhibitors for mild steel in acidic environments [1–8]. All effective inhibitors possess heteroatoms (O, N, S), benzene rings with saturated and unsaturated bonds. The inhibitors usually form a complex on a metal surface, by transferring electrons and forming a coordinate covalent bond during the chemical adsorption. In this way, the metal acts as an electrophile, and the heteroatoms present in the inhibitor act as nucleophilic centres with free electron pairs that are readily available for sharing.

As a result, there exists a need to enlarge economical and ecologically affable inhibitors. In recent years, researchers have had awareness to the progress of drugs as inhibitors to the deterioration of metals in acid media [9–15]. Most of the drugs work effectively in the corrosive blood environment of our body. This led to the motivation of our present study to find out the effect of biotin in 15% hydrochloric acid (HCl) solution for protection of mild steel. Biotin, also known as vitamin H or B7, is a water-soluble B-complex vitamin which is composed of a tetrahydroimidizalone ring fused with a tetrahydrothiophene ring. It is used as a drug for enhancing cell growth, production of fatty acids, and for metabolism of fats and amino acids. The transportation of carbon dioxide and various other metabolic reactions are assisted effectively by biotin. It may also be helpful in maintaining a steady blood sugar level. Hair loss and discoloration of nails are common problems, and biotin is often recommended for strengthening hair and nails. Therefore, biotin is an important ingredient in many cosmetic and skincare products for hair and nails. There is no report on the use of biotin as a corrosion inhibitor in HCl solution.

## 2. Experimental

### 2.1. Materials

**Figure 1.**
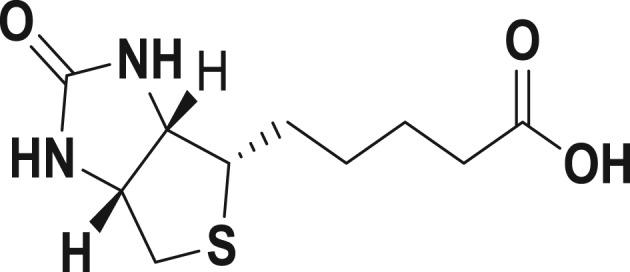
Biotin- IUPAC name 5-[(3a*S*,4*S*,6a*R*)-2-oxohexahydro-1*H*-thieno[3,4-*d*]imidazol-4-yl]pentanoic acid.

Mild steel coupons of rectangular shape (5.0 × 2.5 × 0.25 cm and 30 mm × 3 mm × 3 mm) having the composition (wt %): C 0.17%; Mn 0.46%; Si 0.026%; Cr 0.050%; P 0.012%; Cu 0.135%; Al 0.023%; Ni 0.05%; and balance Fe were used for weight loss studies. Mild steel coupons were abraded with emery paper, washed systematically with double-distilled water and at last degreased with acetone. The hostile solution 15% HCl was prepared by dilution of analytical grade HCl with double-distilled water, and all experiments were carried out in unstirred solutions. Biotin was procured from Ranbaxy Pharmaceuticals Limited and its structure is shown in figure 1. The International Union of Pure and Applied Chemistry (IUPAC) name of biotin is 5-[(3aS,4S,6aR)-2-oxohexahydro-1H-thieno[3,4-d]imidazol-4-yl]pentanoic acid, with molar mass 244.31 g mol^−1^ and melting point 232°C; it is soluble in water.

### 2.2. Procedures

#### 2.2.1. Weight loss measurements

Weight loss experiments were carried out for different concentrations of the inhibitor (50–500 ppm), different immersion times (2–8 h) and different temperatures (308–338 K) in 100 ml of 15% HCl solution. Every sample was weighed by an electronic balance before exposing it to the acid solution (100 ml). After immersion, the samples were cleaned by sodium bicarbonate solution, followed by rinsing with acetone, and the sample was weighed again in order to calculate the inhibition efficiency (*η*%) and the corrosion rate (*C*_R_). The inhibition efficiency and the surface coverage (*θ*) were determined by using the following equations [16]:

2.1 $$\theta =\frac{w_{0}-w_{i}}{w_{0}}$$

and

2.2 $$\eta \%=\frac{w_{0}-w_{i}}{w_{0}}\times 100,$$

where *w*_i_ and *w*_0_ are the weight loss values in the presence and absence of inhibitor, respectively.

The corrosion rate (*C*_R_) of mild steel was calculated using the following relation [17]:

2.3 $$C_{R}\left(\frac{\text{mm}}{y}\right)=\frac{87.6\times w}{atD},$$

where *w* is the corrosion weight loss of mild steel (mg), *a* is the area of the coupon (cm^2^), *t* is the exposure time (h) and *D* is the density of mild steel (g cm^−3^).

#### 2.2.2. Electrochemical measurements

The electrochemical measurements were made using a Gamry Potentiostat/Galvanostat (Model G-300) in a three-electrode cell assemblage at room temperature (308 K). Mild steel of 1.0 × 1.0 cm was used as the working electrode; a platinum electrode was used as an auxiliary electrode; and a standard calomel electrode (SCE) was used as the reference electrode. Gamry applications include the software DC 105 for corrosion and EIS 300 for electrochemical impedance spectroscopy (EIS) measurements and Echem Analyst version 5.50 software packages for data fitting [18]. EIS measurements were carried out in a frequency range from 100 kHz to 0.00001 kHz under potentiodynamic conditions, with an amplitude of 10 mV peak-to-peak, using AC signal at *E*_corr_ [19]. Prior to the electrochemical measurement, a stabilization period of 30 min was allowed, which was proved to be sufficient to attain a stable value of *E*_corr_ [20]. The charge transfer resistance values were obtained from the diameter of the semicircles of the Nyquist plots. The inhibition efficiency of the inhibitor was found out from the charge transfer resistance values using the following equation:

2.4 $$\eta \%=\frac{R_{\mathrm{ct}}^{i}-R_{\mathrm{ct}}^{0}}{R_{\mathrm{ct}}^{i}}\times 100,$$

where $R_{\mathrm{ct}}^{0}$ and $R_{\mathrm{ct}}^{i}$ are the charge transfer resistance in the absence and presence of inhibitor, respectively.

Tafel curves were obtained by changing the electrode potential automatically from −250 to +250 mV versus the corrosion potential (*E*_corr_) at a sweep rate of 1 mV s^−1^. The inhibition efficiency was evaluated from the measured *I*_corr_ values using the relationship

2.5 $$\eta \%=\frac{I_{\mathrm{corr}}^{0}-I_{\mathrm{corr}}^{i}}{I_{\mathrm{corr}}^{0}}\times 100,$$

where $I_{\mathrm{corr}}^{0}$ and $I_{\mathrm{corr}}^{i}$ are the corrosion currents in the absence and presence of inhibitor, respectively. All electrochemical measurements were done in unstirred and non-deaerated solutions [21].

### 2.3. Surface characterization

#### 2.3.1. Contact angle measurement

Mild steel samples were exposed to the drops of the acid solution with and without inhibitor to detect the contact angle using the DSA100 Kruss instrument. Mild steel samples were rinsed with water and acetone to remove any sort of contamination on the surface [22].

#### 2.3.2. Scanning electrochemical microscopy

The scanning electrochemical microscopy (SECM) technique can reveal important changes on the metal surface. The set-up is very similar to the electrochemical measurement as the three-electrode assembly is used to detect the current flow through a microelectrode immersed in an electrolytic solution. An electrochemical work station of the CHI900C model was used with mild steel as the working electrode, a reference electrode and a platinum counter electrode for all the tests [23,24].

#### 2.3.3. Scanning electron microscopy

The morphological changes of the mild steel sample in the absence and presence of inhibitors were analysed by the scanning electron microscopy (SEM) technique. In this, the mild steel sample was immersed in the test solution both in the absence and presence of 100, 200 and 500 ppm concentrations of inhibitor at 308 K for 6 h. After that, the metal sample was taken out, cleaned with double-distilled water and dried at room temperature. The instrument model used for SEM studies was TESCAN VEGA II XMH. Gold was sprayed on the metal surface for better conductivity and good quality of images [25].

#### 2.3.4. Atomic force microscopy

The coupons of mild steel after immersion in the test solution in the presence and absence of biotin were taken for atomic force microscopy (AFM) studies using the NT-MDT SOLVER Next AFM/STM instrument. The scan size of each sample used in AFM is 10 µm × 10 µm.

### 2.4. Quantum chemical calculations

In the present case, all quantum chemical studies have been carried out with the help of DFT/B3LYP methods using a 6-31G (d, p) basis set using the Gaussian 09 program package. It is well known that the corrosion process takes place in the aqueous phase, so it is computationally suitable to include the effect of solvent, and thus all quantum calculations were carried out in the aqueous phase using self-consistent reaction field (SCRF) theory, with the polarized continuum model (PCM). The neutral and protonated forms of inhibitor molecules were studied and the energy of HOMO and LUMO orbitals and energy (Δ*E*) were determined [26]. Fukui parameters were calculated to detect the electrophilic and nucleophilic sites of the inhibitor molecules participating in bond formation.

## 3. Results and discussion

### 3.1. Weight loss studies

#### 3.1.1. Effect of inhibitor concentration

**Figure 2.**
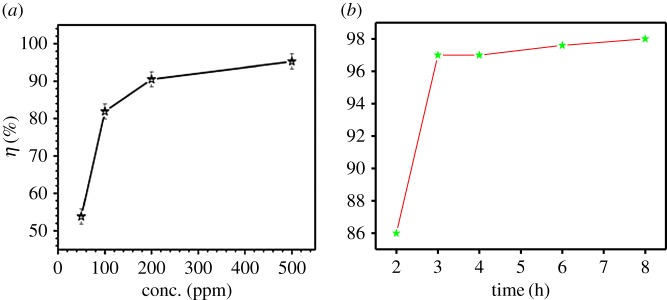
Variation of the inhibition efficiency in 15% HCl on mild steel with (*a*) different concentrations of biotin and (*b*) different immersion times.

Figure 2*a* represents the effect of inhibitor concentration on inhibition efficiency in HCl. The inhibitor showed maximum inhibition efficiency of 98% in HCl at a concentration of 500 ppm. The values of percentage inhibition efficiency (*η*%) and corrosion rate (*C*_R_) obtained from the weight loss method at different concentrations of biotin at 308 K are summarized in table 1. From table 1 it is clear that increase in inhibitor concentrations caused a decrease in the weight loss as well as corrosion rate of mild steel [27].

**Table 1.** Weight loss parameters for biotin on mild steel at different concentrations.

inhibitor	concentration (mg l^−1^)	corrosion rate (mg cm^−2^ h^−1^)	surface coverage (*θ*)	*η* (%)
blank	0	7.0	—	—
biotin	50	3.3	0.537	53.7
	100	1.3	0.819	81.9
	200	0.6	0.907	90.7
	500	0.3	0.953	95.3

#### 3.1.2. Effect of time

To assess the stability of the inhibitive behaviour of inhibitor on a timescale, weight loss measurements were performed for 2–8 h of immersion time at 308 K. Inhibition efficiencies were plotted against immersion time as seen from figure 2*b*. The increase in inhibition efficiency from 2 to 8 h reflects the strong adsorption of constituents present in the biotin compound on the mild steel surface, resulting in a more protective layer formed at the mild steel/HCl solution interface. From the figure it is observed that inhibition efficiency increases from 86.5% to 98.0% with time [6].

#### 3.1.3. Effect of temperature

The effect of temperature on the inhibition efficiency of the biotin for mild steel in 15% HCl solution at temperatures ranging from 308 to 338 K was investigated by weight loss measurements. The results obtained are given in table 2. It is observed that as the temperature increases from 308 to 338 K, inhibition efficiency decreases while the corrosion rate increases. This behaviour can be explained on the basis that the increase in temperature causes desorption of the inhibitor molecules from the surface of mild steel [28].

**Table 2.** Effect of temperature on inhibition efficiency at a 500 ppm concentration of biotin.

inhibitor	*T* (*K*)	*η* (%)
blank	308	95.3
	318	84.8
	328	66.9
	338	38.8

#### 3.1.4. Thermodynamic activation parameters

**Figure 3.**
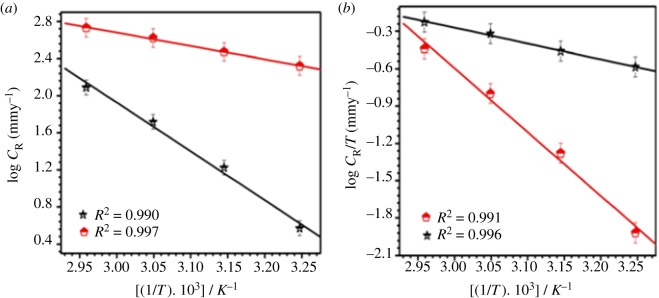
(*a*) Arrhenius plot of log *C*_R_ versus 1000/*T*. (*b*) Transition state plot of log *C*_R_/*T* versus 1000/*T* for mild steel in 15% HCl in the absence and the presence of biotin.

To find the activation parameters of the inhibition process for mild steel in 15% HCl solution, weight loss measurements were performed at a temperature range 308–338 K in the absence and presence of biotin. A plot of the logarithm of the corrosion rate (mg cm^−2^ h^−1^) of mild steel versus 1000/*T* gave a straight line as shown in figure 3*a*. According to the Arrhenius equation [29]:

3.1 $$C_{R}=A\;\exp\left(\frac{-E_{a}}{RT}\right),$$

where *E*_a_ is the apparent activation energy for the corrosion of mild steel in 15% HCl solution, *R* is the gas constant, *A* is the Arrhenius pre-exponential factor and *T* is the absolute temperature. The values of *E*_a_ obtained from the slope of the line (figure 3*a*) are given in table 3.

**Table 3.** Thermodynamic parameters for the adsorption of biotin on mild steel in 15% HCl at a 500 ppm concentration.

	*K*_ads_	$-\Delta G_{\mathrm{ads}}^{o}$	$\Delta H_{a}^{o}$	$\Delta S_{a}^{o}$
inhibitor	(10^4^ M^−1^)	(kJ mol^−1^)	(kJ mol^−1^)	(JK^−1^ mol^−1^)
blank	—	—	24.26	−129.96
biotin	2.38	36.09	98.26	142.09

The straight lines were obtained according to the transition state equation:

3.2 $$C_{R}=\frac{RT}{Nh}\;\exp\;\left(\frac{\Delta S^{*}}{R}\right)\;\exp\;\left(-\frac{H^{*}}{RT}\right),$$

where *N* is the Avogadro's number, *h* is the Planck's constant, ΔH* is the enthalpy of activation and ΔS* is the entropy of activation [30].

Figure 3*b* shows that a plot of log (*C*_R_/*T*) versus 1000/*T* gives a straight line with a slope of (−ΔH*/2.303 *R*) and an intercept of log (*R*/*Nh* + ΔS*/2.303 *R*) from which the values of ΔH*and ΔS*are calculated and are given in table 3.

Table 3 shows that the value of enthalpy of activation is positive and higher in the presence of inhibitor. The positive sign of ΔH*reflects the endothermic nature of the mild steel dissolution process, suggesting that the dissolution of mild steel is slow [31,32]. The entropy of activation ΔS* is higher in the presence of inhibitor than that in the absence of the inhibitor. This could be explained considering the adsorption of organic inhibitor molecules from the aqueous solution as a quasi-substitution process between the inhibitor in the aqueous phase [Inh_(sol)_] and water molecules at the electrode surface [H_2_O_(ads)_] [33]. In this situation, the adsorption of inhibitor is accompanied by desorption of water molecules from the surface. The thermodynamic values obtained are the algebraic sum of the adsorption of inhibitor molecules and desorption of water molecules. Therefore, the gain in entropy is attributed to the increase in solvent entropy [34]. The positive values of ΔS* means that the adsorption process is accompanied by an increase in entropy, which is the driving force for the adsorption of inhibitor onto the mild steel surface [35].

#### 3.1.5. Adsorption considerations

The standard free energy of adsorption ($\Delta G_{\mathrm{ads}}^{o}$) at different temperatures is calculated from the equation

3.3 $$\Delta G_{\mathrm{ads}}^{o}=-RT\ln(55.5K_{\mathrm{ads}}),$$

where the value 55.55 is the concentration of water in solution expressed in Moles [36–38], and *K*_ads_ is the equilibrium adsorption constant and is given by

3.4 $$K_{\mathrm{ads}}C=\frac{\theta }{1-\theta },$$

where *θ* is degree of surface coverage of the mild steel surface and *C* is the molar concentration of the inhibitor. The values of *K*_ads_ and $\Delta G_{\mathrm{ads}}^{o}$ for mild steel in 15% HCl solution in the presence of 500 ppm biotin is given in table 3.

The negative values of $\Delta G_{\mathrm{ads}}^{o}$ ensure the spontaneity of the adsorption process and stability of the adsorbed layer on the steel surface. Generally, values of $\Delta G_{\mathrm{ads}}^{o}$ around −20 kJ mol^−1^ or lower are consistent with physisorption, while around −40 kJ mol^−1^ or higher values with chemisorption [32,39–41]. The calculated value in the present study is −36.09 kJ mol^−1^ (table 3). This indicates that biotin is adsorbed physically [42].

**Figure 4.**
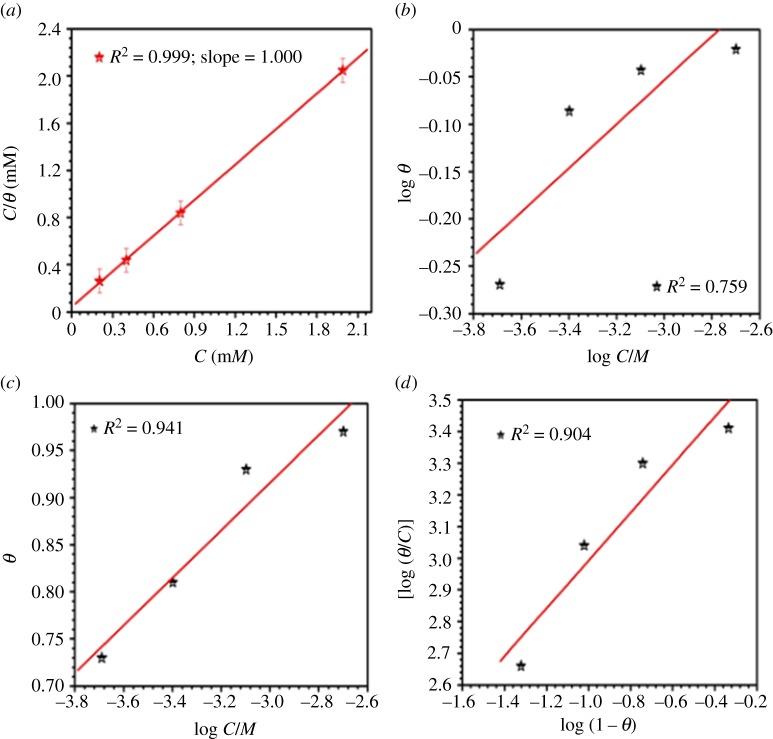
Adsorption plots using (*a*) Langmuir, (*b*) Freundlich, (*c*) Temkin, and (*d*) Flory-Huggins isotherms.

Surface coverage (*θ*) values were tried to fit into the Langmuir, Freundlich, Temkin and Flory- Huggins isotherms (figure 4*a–d*), and the correlation coefficient (*R*^2^) values were used to establish the best fit isotherm. The superlative outcome was obtained for the Langmuir adsorption isotherm. A straight line was obtained on plotting *C*/*θ* versus *C* as shown in figure 4*a*. It suggested that the adsorption of the inhibitor at the metal/solution interface follows Langmuir's adsorption isotherm.

### 3.2. Electrochemical measurements

#### 3.2.1. Open circuit potential versus time

**Figure 5.**
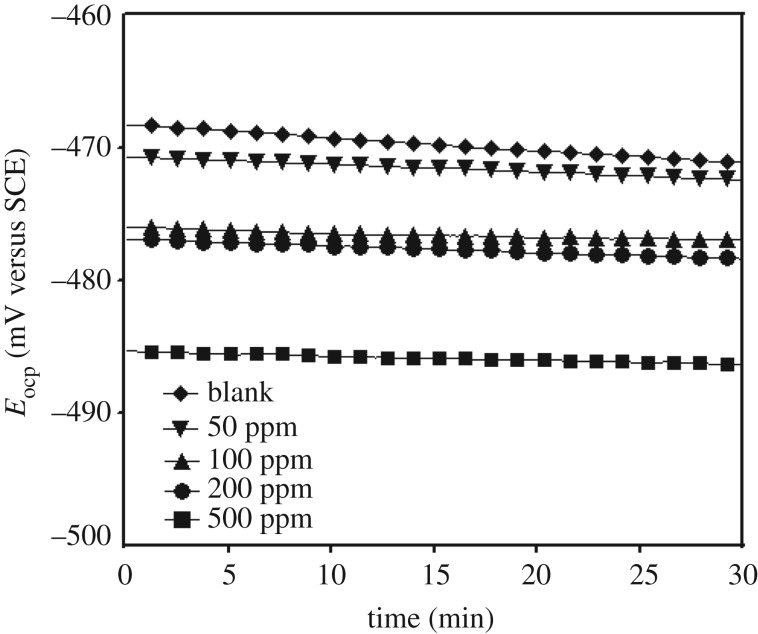
OCP plots of mild steel in the absence and presence of biotin in 15% HCl solution.

**Figure 6.**
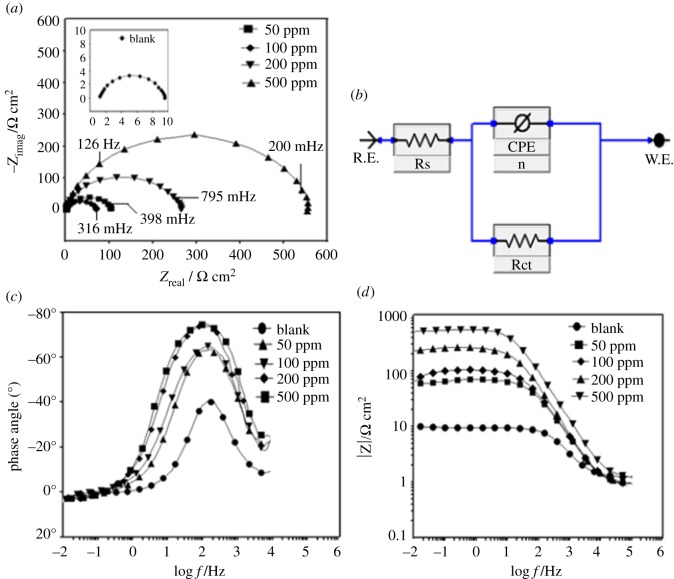
Electrochemical impedance parameters (*a*) Nyquist plots, (*b*) Bode-modulus plots, (*c*) phase angle plots for biotin in 15% HCl solution, and (*d*) electrochemical equivalent circuit used to fit the impedance spectra.

Prior to running potentiodynamic polarization and EIS, it is necessary to maintain the stability of the open circuit potential (OCP). Figure 5 depicts the variation of the OCP of the steel electrode with time in 15% HCl solution in the absence and presence of different concentrations of biotin at 308 K. In the absence of inhibitors, that the steady-state values of OCP are more negative than the immersion potential (*E*_ocp_ at *t* = 0) suggests that before the steady-state condition is achieved, the pre-immersion, air-formed oxide film on the electrode has to dissolve [43]. It is obvious from figure 6 that addition of studied concentrations of biotin to 15% HCl solution shifts the steady-state potential (*E*_corr_) to more negative values without changing the general features of the E–t curves, indicating that they catalyse the oxide film dissolution. These results may be interpreted on the basis of formation of stable Fe (II) complexes with the N, S-containing ligands [43,44].

#### 3.2.2. AC technique: electrochemical impedance spectroscopy

AC impedance results of the mild steel/HCl interface obtained in the absence and presence of various concentrations of biotin in the form of Nyquist plots are shown in figure 6*a*. Figure 6*a* suggests that the impedance spectra exhibit a depressed capacitive loop, which has the centre below the real axis, indicating the roughness and the inhomogeneities at the mild steel electrode. Also the diameter of the capacitive loop increases with the increase in the concentration of inhibitor [45–47].

The simplest equivalent model used to fit the data is shown in figure 6*b*. This consists of the solution resistance, *R*_s_, which is in series with the parallel combination of the constant phase element (CPE) and the charge transfer resistance (*R*_ct_). Mathematically, a CPE's impedance is given by [48]

3.5 $$Y_{\mathrm{CPE}}=Y_{0}{\left(j\omega \right)}^{n},$$

where *Y*_0_ is the amplitude comparable to a capacitance, *j* is the imaginary unit, *ω* is the angular frequency (*ω* = 2*πf*, the frequency in Hz) and *n* is the phase shift, which gives details about the degree of surface inhomogeneity. The CPE can be expressed by the values of *n* if resistance (*n* = 0, *Y*_0_ = *R*), capacitance (*n* = 1, *Y*_0_ = *C*), inductance (*n* = −1, *Y*_0_ = *L*) and Warburg impedance (*n* = 0.5, *Y*_0_ = *W*) [49–51]. The impedance data are given in table 3.

Table 4 shows that the addition of the biotin in 15% HCl increases the inhibition efficiency, charge transfer resistance and decreases the double layer capacitance (*C*_dl_) given as follows [52]:

3.6 $$C_{\mathrm{dl}}=\frac{\varepsilon \varepsilon_{0}}{d}A,$$

where *ε*_0_ is the vacuum dielectric constant, *ε* is the local dielectric constant, *d* is the thickness of the double layer and *A* is the surface area of the electrode. Decrease in the capacitance leading to decrease in the local dielectric constant or increase in the thickness of the electrical double layer strongly suggests that the inhibitor molecules are adsorbed at the metal/solution interface [30]. Figure 6*c*,*d* shows the Bode and phase angle plots recorded for the mild steel electrode immersed in 15% HCl in the absence and presence of various concentrations of biotin at its open circuit potential. The values of Bode impedance magnitude (*S*) and maximum phase angles (*a*°) are listed in table 5. In the intermediate frequency region, a linear relationship between log |*Z*| and log *f*, where the slope is near −1 and the phase angle tends to become −90°, can be observed. An ideal capacitive response would result in a slope of −1 and a phase angle of −90° [53]. In our case, a linear relationship between log |*Z*| and log *f,* with the slope near −0.720 and the phase angle approaching 80°, in the intermediate-frequency region has been observed. The Bode phase angle plots show a single maximum (one time constant) at intermediate frequencies, and broadening of this maximum in the presence of biotin accounts for the formation of a protective layer on the electrode surface [32,54].

**Table 4.** Electrochemical impedance parameters for mild steel dipped in 15% HCl in the absence and presence of different concentrations of biotin.

solution	*R*_s_ (**Ω** cm^2^)	*R*_ct_ (**Ω** cm^2^)	*n*	*Y*_°_ (**Ω**^−1^s^n^/cm^2^)	*τ* (sec)	*C*_dl_ (μF cm^2^)	*χ*^2^	*η* %	surf. coverage *θ*
1 M HCl	1.2	18	0.828	248	496.2	79.8	0.0006	—	—
biotin 50 ppm	2.9	66	0.830	115	157.0	40.8	0.0008	73	0.73
biotin 100 ppm	1.8	94	0.839	108	49.4	36.1	0.0033	81	0.81
biotin 200 ppm	2.6	255	0.859	71	19.6	27.9	0.0019	93	0.93
biotin 500 ppm	2.1	547	0.880	34	7.3	12.5	0.0011	97	0.97

**Table 5.** The slopes of the Bode impedance magnitude plots at intermediate frequencies (*S*) and the maximum phase angles (*α*) for mild steel in 15% HCl in the absence and presence of biotin.

conc. (ppm)	−*S*	−*α*°
1 M HCl	0.429	38.4
biotin 50 ppm	0.482	61.3
biotin 100 ppm	0.531	62.4
biotin 200 ppm	0.629	78.7
biotin 500 ppm	0.720	79.2

#### 3.2.3. Potentiodynamic polarization

**Figure 7.**
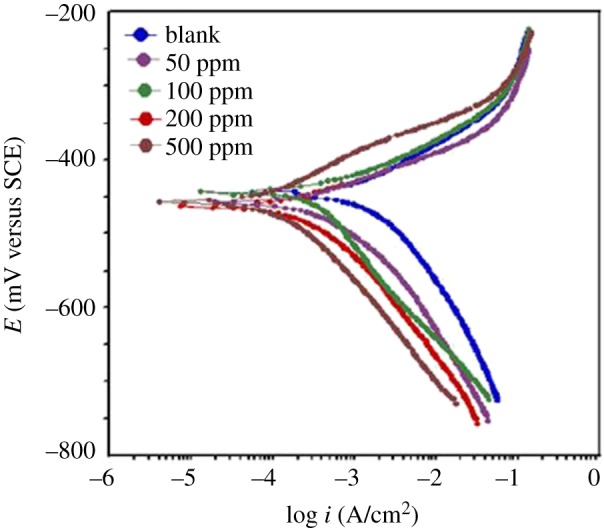
Polarization curves in the absence and presence of different concentrations of biotin in 15% HCl solution.

Polarization curves for mild steel at various concentrations of biotin are shown in figure 7. It is observed that both the cathodic and anodic reactions are suppressed with the addition of biotin, which suggests that it inhibits both anodic dissolution and cathodic hydrogen evolution reaction. Electrochemical corrosion parameters i.e. corrosion potential (*E*_corr_) and corrosion current density (*I*_corr_), obtained from the Tafel extrapolation of the polarization curves along with the inhibition efficiency are given in table 6. There was no significant change in the *E*_corr_ values in the presence of biotin, which suggests that it is a mixed-type inhibitor [55–57].

**Table 6.** Polarization parameters for mild steel in 15% HCl at a scan rate of 1 mV s^−1^ in the absence and presence of different concentrations of biotin.

	Tafel data					
conc. (ppm)	*E*_corr_ (V versus SCE)	*I*_corr_ (µA cm^−2^)	*b*_a_ (mV d^−1^)	−*b*_c_ (mV d^−1^)	*η* (%)	surface coverage *θ*
1 M HCl	−0.421	870	75	116	—	—
biotin 50 ppm	−0.432	297	81	102	66	0.66
biotin 100 ppm	−0.445	98	70	99	89	0.89
biotin 200 ppm	−0.460	37	66	93	96	0.96
biotin 500 ppm	−0.449	29	78	109	97	0.97

The values of *β*_c_ did not change significantly with increasing biotin concentrations when compared with the blank, which indicated that the inhibitor is not affected by the hydrogen reduction mechanism [58]. Compared with the blank, the anodic curves of the working electrode in the acid solution containing biotin shifted obviously to the direction of the current reduction, which implied that the biotin could also suppress the anodic reaction. Only when the change in the *E*_corr_ value was more than 85 mV, a compound could be recognized as an anodic- or a cathodic-type inhibitor [32,59]. The largest displacement of *E*_corr_ was about 39 mV (table 6). Therefore, biotin might act as a mixed-type inhibitor. A lower *I*_corr_ value for biotin solutions implies that the rate of electrochemical reactions was reduced owing to the formation of a barrier layer over the mild steel surface by the biotin molecules [60].

### 3.3. Contact angle measurement

**Figure 8.**
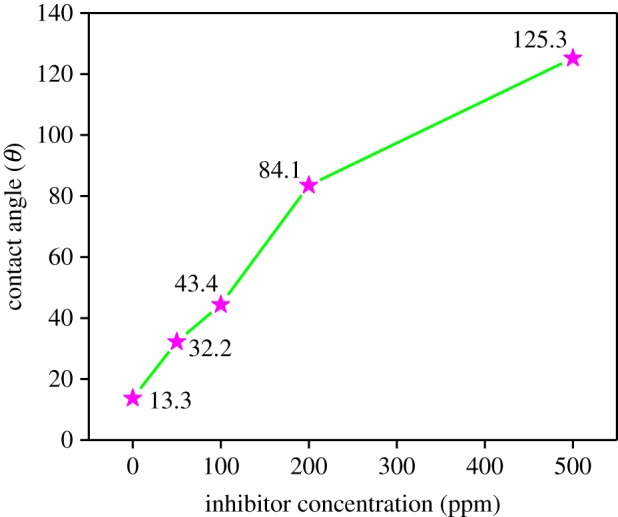
Variation of the contact angle with increase in inhibitor concentration.

A baseline test without a corrosion inhibitor was carried out first after which the corrosion inhibitor was injected on the mild steel sample surface and the concentration was increased in steps. For each concentration of corrosion inhibitor the contact angle measurements were repeated three times. The contact angle of steel surfaces without inhibitor was measured as 13.3° in the 15% HCl solution, meaning that the wettability of the steel surface bestows hydrophilicity (favours water) [61]. With the addition of inhibitor, the contact angle increased from 32.2°, 43.4°, 84.1° to 125.3°, suggesting that the steel surface became hydrophobic (does not favour water) as is evident from figure 8. This confirms the formation of a hydrophobic layer on the steel surface in the presence of inhibitor.

### 3.4. Scanning electrochemical microscopy

**Figure 9.**
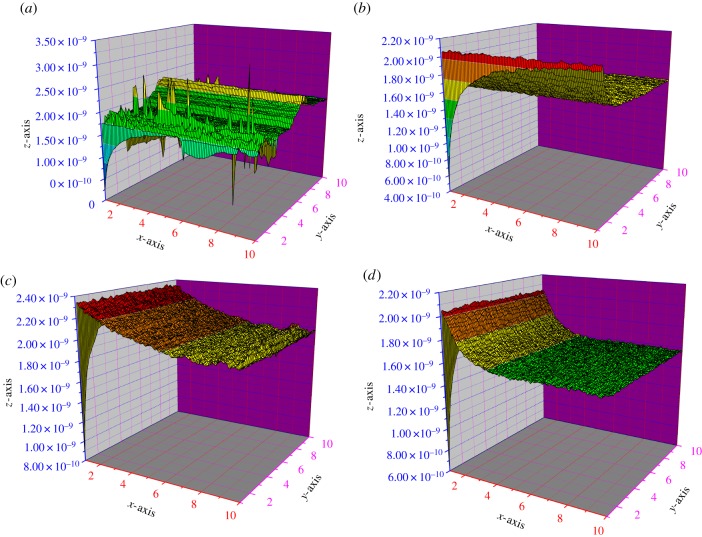
SECM images of mild steel exposed with (*a*) 15% HCl solution *x*-axis (*b*) biotin 500 ppm *x*-axis (*c*) 15% HCl solution *y*-axis (*d*) biotin 500 ppm *y*-axis.

The SECM tests were performed in the AC-amperometry mode to obtain the three-dimensional (3D) figures of the metal surface [62,63]. Figure 9*a–d* shows the *x*-axis and *y*-axis images of the metal surface as visualized by SECM [64–66]. A lower current is observed when the tip of the probe is brought near the metal surface with the biotin film (insulating surface). This may be attributed to the insulating film, which blocks the diffusion of oxygen towards the tip as shown in figure 9*b–d* [67]. On the other hand, the current increases when the tip of the probe is brought near the metal surface without inhibitor (conducting surface). This may be attributed to the presence of the redox mediator that is revived at the surface as shown in figure 9*a–c* [68]. The mild steel surface remains conductive when devoid of inhibitor, and insulating with the inhibitor, which can be confirmed by the enhancement in the current (conducting) and by reduction in the current (insulating) [69].

### 3.5. Scanning electron microscopy

**Figure 10.**
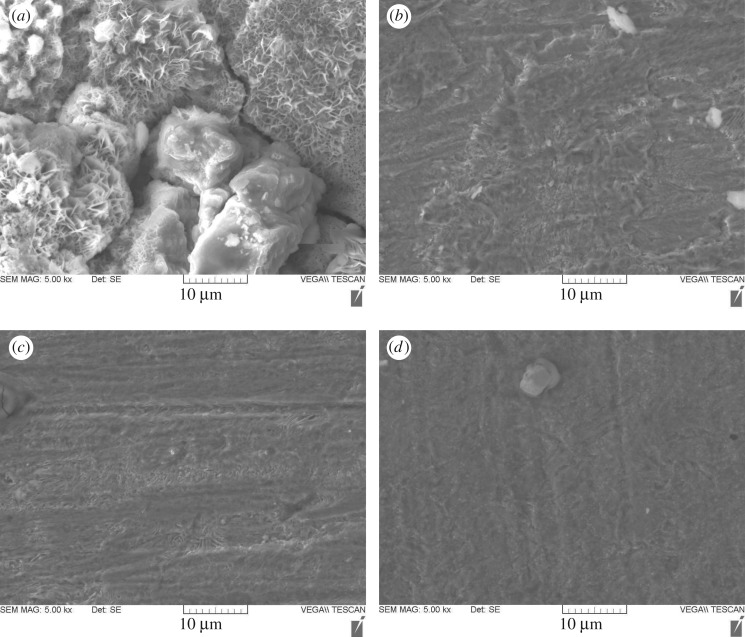
SEM images of mild steel exposed with (*a*) 15% HCl solution, (*b*) biotin 100 ppm, (*c*) biotin 200 ppm, and (*d*) biotin 500 ppm.

SEM photographs were taken to show that the corrosion inhibition is owing to the formation of an adsorptive film on the steel surface. The morphology of the metal in figure 10*a* showed a corroded/rough surface in the absence of inhibitors, and the surface is strongly damaged. However, in the presence of biotin, the surface corrosion of mild steel is remarkably decreased. The surface was less corroded and smooth for 100, 200 and 500 ppm, as shown in figure 10*b–d* [70]. These results prove that a protective film was formed on the metal surface which can effectively protect mild steel samples from a corrosive environment.

### 3.6. Atomic force microscopy

**Figure 11.**
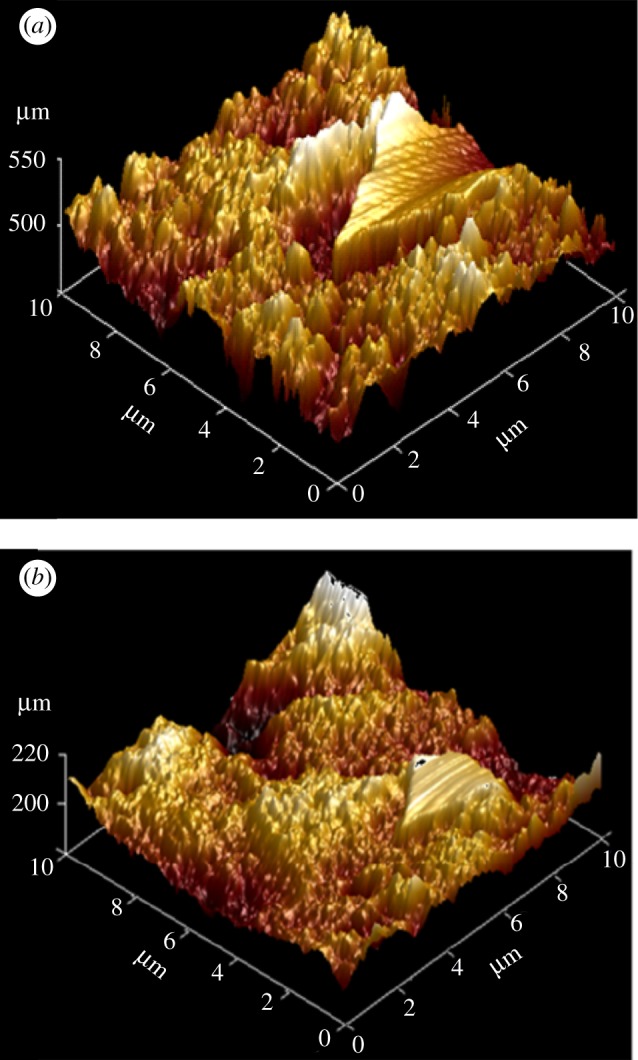
Atomic force microscopic images of (*a*) blank 15% HCl and (*b*) 500 ppm biotin.

The 3D AFM morphologies in the absence and presence of inhibitors are given in figure 11*a*,*b*. The parameters calculated are *R*_q_ (root-mean-square roughness), *R*_a_ (average roughness) and *R*_Δq_ (root mean square slope of roughness).

Figure 11*a* represents a highly corroded surface in the absence of inhibitor, but as the inhibitor is added, the surface morphology becomes smooth (figure 11*b*), suggesting inhibitor film formation over the mild steel surface. Also from table 7, it could be observed that the values of *R*_q_ and *R*_a_, are large in the absence of inhibitor, revealing greater surface roughness. But in the presence of inhibitor, all the calculated parameters values are reduced, which confirms that the surface becomes smoother and this smoothness occurs owing to the formation of a compact protective film of inhibitor.

**Table 7.** The surface roughness parameters obtained by AFM tests.

sample	*R*_q_ (μm)	*R*_a_ (μm)	*R*_Δq_
blank	151.016	122.410	0.662
biotin	25.864	19.714	0.930

The last parameter *R*_Δq_ represents the corrosion resistance behaviour of the metal. Table 7 revealed that in the absence of inhibitor the *R*_Δq_ value is less compared to that in the presence of inhibitor. These data justify that in the absence of inhibitor the mild steel is going to corrode at a greater extent than in the presence of inhibitor. This reduction in corrosion in the presence of inhibitor is owing to the formation of a protective film of inhibitor over the mild steel surface, which checks the corrosion process.

### 3.7. Quantum chemical calculations

**Figure 12.**
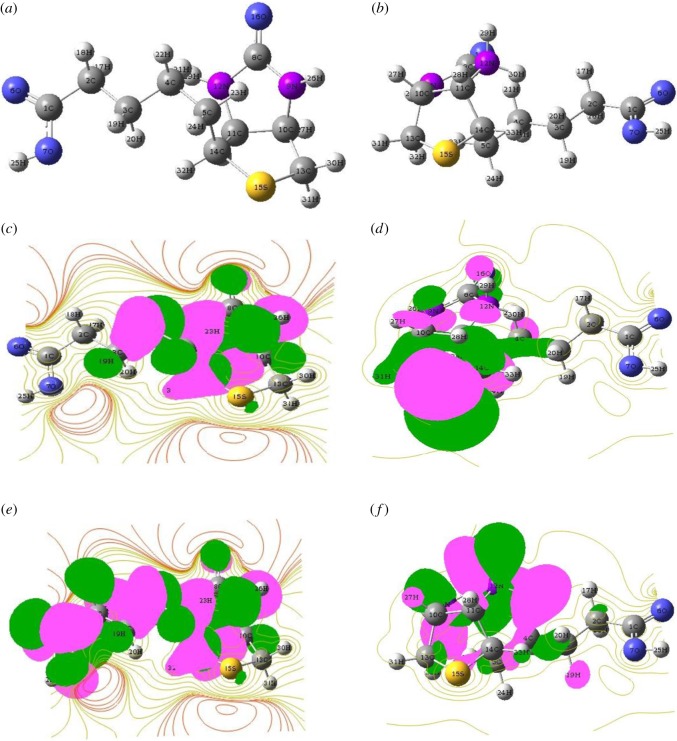
(*a*) Neutral optimized molecular structure, (*b*) Protonated optimized molecular structure, (*c*) HOMO for the neutral molecule, (*d*) HOMO for the protonated molecule, (*e*) LUMO for the neutral molecule, and (*f*) LUMO for the protonated molecule.

The frontier molecular orbitals (FMOs) of inhibitor molecules and the metallic surface undergo donor–acceptor-type interaction. The optimized structure and the HOMO/LUMO distribution of neutral inhibitors are represented in figure 12*a–f*. The HOMO and LUMO are distributed over the heteroatoms and phenyl rings, respectively.

To study the chemical reactivity of inhibitor molecules, the analysis of the energy of the highest occupied molecular orbital (*E*_HOMO_) and the energy of the lowest unoccupied molecular orbital (*E*_LUMO_) has been studied. According to FMO theory, *E*_HOMO_ is related to the capacity of a molecule to donate electrons, and the higher the value of *E*_HOMO,_ higher would be the electron-donating tendency of the molecule [71–73]. Higher *E*_LUMO_ represents the ability of the molecule to accept electrons, whereas lower values of *E*_LUMO_ indicates more probably the ability of the molecule to accept electrons [74]. The values of the energy of the highest occupied molecular orbital (*E*_HOMO_), energy of the lowest unoccupied molecular orbital (*E*_LUMO_) and energy gap i.e. Δ*E* = (*E*_LUMO_ − *E*_HOMO_) are tabulated in table 8. The energy gap Δ*E* is also an important parameter and it represents the reactivity of the inhibitor molecule towards the adsorption on the metal surface. The lower the value of Δ*E,* the higher would be the adsorption tendency. Thus the results obtained from quantum chemical studies are in accordance with the experimental results.

**Table 8.** Parameters obtained from quantum chemical calculations for mild steel in 15% HCl.

	*E*_HOMO_ (eV)	*E*_LUMO_ (eV)	Δ*E* (eV)	Δ*N*
neutral	−5.984	0.254	6.238	0.313
protonated	−6.707	−1.354	5.353	0.182

#### 3.7.1. Protonated inhibitor

The organic molecules have a tendency to undergo protonation in aqueous medium and in this form they also get adsorbed on the metal surface. So, it becomes necessary to study the chemistry of adsorption of protonated molecules. To find out the most preferred site for protonation, we have calculated the proton affinity (PA) at the sites N_9_ and N_12_ by using the following equation:

3.7 $$E_{\mathrm{protonation}/\mathrm{aqueous}}=E_{\mathrm{pro}(\mathrm{aqueous})}--E_{\mathrm{neutral}(\mathrm{aqueous})}-E_{H+(\mathrm{aqueous})},$$

where *E*_pro(aqueous),_ *E*_neutral(aqueous)_ and *E*_H_ _+_ _(aqueous)_ are the total energies of the protonated inhibitor, neutral inhibitor and proton in the aqueous phase, respectively. The site which has the most negative value of PA would be the preferred site for protonation. In the present case, N_12_ has the most negative value of PA, thus it would be the preferred site for protonation (table 9).

**Table 9.** Mulliken charges on heteroatoms and proton affinity values.

					PA (kcal mol^−1^)			
inhibitors	O_6_	O_16_	N_9_	N_12_	N_12_	N_9_	O_6_	O_16_
ADP	−0.520	−0.483	−0.569	−0.583	−503.552	−497.607	478.782	473.134

An observation of table 8 reveals that after protonation, the *E*_HOMO_ value of the inhibitor shifted more towards the negative side compared to that of the neutral inhibitor. It suggests that after protonation, the electron-donating capability of inhibitors decreased. It is also noted that the *E*_LUMO_ value of protonated inhibitor shifted more towards the negative side when compared to that of the neutral inhibitor, which suggests that the protonated inhibitors have a higher electron-accepting capability compared to the neutral form.

As we know that in the aqueous phase, both neutral and protonated forms of inhibitor molecules would get adsorbed on the metal surface, a competition arises among the neutral and protonated molecules as to which one would be preferentially adsorbed. Hence, by comparing the Δ*E* values of neutral and protonated inhibitor molecules, the adsorption capability can be solved (table 8) and because the value of Δ*E* is lower for inhibitor in a protonated form than in a neutral form, which reveals that the protonated species can easily be adsorbed on the mild steel surface than the neutral [75].

The fraction of electrons transferred (Δ*N*) from the inhibitor molecules to the metal surface are calculated using the following equation [74]:

$$\Delta N=\frac{\phi -\chi_{\mathrm{inh}}}{2(\eta_{\mathrm{Fe}}+\eta_{\mathrm{inh}})},$$

where, *χ*_inh_, *η*_Fe_ and *η*_inh_ are the respective electronegativity and hardness values of iron and inhibitor molecules, respectively. The *η*_Fe_ of iron is 0 and *ϕ* is the work function of iron and its value is 4.82 [76]. The value of Δ*N* for neutral inhibitor is greater than that for the protonated one. Thus, inhibitor in a neutral form transfers more electrons when compared with that in a protonated form.

#### 3.7.2. Fukui index analysis

The maximum threshold values of $f_{k}^{+}$ and $f_{k}^{-}$ represent the nucleophilic and electrophilic sites of the inhibitor molecules and they are calculated by the Fukui indices analysis [77]. The electron-donating capacity of inhibitor molecules are measured by high values of $f_{k}^{-}$ and the electron-accepting ability of inhibitor molecules are measured by high values of $f_{k}^{+}$, and are tabulated in table 10; it is revealed that N, O, C-atoms of the inhibitor are the most susceptible sites for electron acceptance or donation. The most susceptible sites for electrophilic attacks i.e. electron donation are C(2), C(3), C(4), C(5), C(8), N(9), C(10), C(11), N(12), C(13), C(14), S(15), O(16) atoms, and the favourable sites for electron acceptance ($f_{k}^{+}$) i.e. nucleophilic attacks are C(1), C(2),C(3), C(4), C(5), O(6), O(7), C(8), C(11), N(12), C(14), respectively. After the analysis, it can be concluded that the heterocyclic moieties along with the phenyl ring are the reactive sites which are more responsible for donor–acceptor interactions and thus facilitate the adsorption of inhibitors over the metallic surfaces.

**Table 10.** Calculated Fukui functions for the studied inhibitor molecules in neutral form.

atoms	$f_{k}^{-}$	$f_{k}^{+}$
C1	0.0000	0.7140
C2	0.0004	−0.0324
C3	0.0007	0.0018
C4	0.0049	0.0008
C5	0.0199	−0.0001
O6	0.0000	0.1395
O7	0.0000	0.0599
C8	0.0011	0.0001
N9	0.0030	0.0000
C10	0.0030	0.0000
C11	0.0012	0.0001
N12	0.0005	0.0006
C13	−0.0533	0.0000
C14	−0.0510	0.0001
S15	1.0141	0.0000
O16	0.0007	0.0000

## 4. Conclusion

(i) The results obtained from gravimetric and electrochemical studies reveals that the inhibition efficiency of inhibitor increases with increase in inhibitor concentration.
(ii) A linear fit result (*R*^2^ = 0.99915) was obtained for the Langmuir adsorption isotherm and was a typical chemisorption.
(iii) Polarization measurements revealed that the inhibitor was of mixed type as both the anodic and cathodic processes were hindered.
(iv) Surface studies confirmed the mitigation of corrosion by biotin through formation of a protective layer.
(v) Quantum chemical calculation reveals that protonated species can more easily be adsorbed on the mild steel surface than the neutral species.

## Data accessibility

Our data are deposited at Dryad: http://datadryad.org/review?doi=doi:10.5061/dryad.1d3m8 [78].
